# Serum Extracellular Vesicle-Associated GULP1 Is a Key Indicator of Hepatocellular Carcinoma

**DOI:** 10.32604/or.2025.070392

**Published:** 2026-01-19

**Authors:** Hyung Seok Kim, Ju A Son, Minji Kang, Soon Sun Kim, Geum Ok Baek, Moon Gyeong Yoon, Se Ha Jang, Dokyung Jung, Ji Eun Han, Jae Youn Cheong, Jung Woo Eun

**Affiliations:** 1 Department of Biochemistry, Kosin University College of Medicine, Seo-gu, Busan, 49267, Republic of Korea; 2 Department of Molecular Diagnostics, Ugenecell, Songpa-gu, Seoul, 05557, Republic of Korea; 3 Department of Gastroenterology, Ajou University School of Medicine, 164 Worldcup-ro, Yeongtong-gu, Suwon, 16499, Republic of Korea; 4 Department of Biomedical Sciences, Ajou University Graduate School of Medicine, 164 Worldcup-ro, Yeongtong-gu, Suwon, 16499, Republic of Korea; 5 Department of Molecular Medicine, Kyungpook National University School of Medicine, 680 Gukchaebosang-ro, Daegu, 41944, Republic of Korea

**Keywords:** Extracellular vesicles, GULP PTB domain-containing engulfment adaptor 1, hepatocellular carcinoma, liquid biopsy, biomarker

## Abstract

**Objectives:**

Early detection of hepatocellular carcinoma (HCC) is a significant challenge due to the limited sensitivity of alpha-fetoprotein (AFP). This study aimed to assess serum-derived extracellular vesicle-encapsulated GULP PTB domain-containing engulfment adaptor 1 (*EV-GULP1*) as a novel, noninvasive biomarker for HCC detection and prognosis, leveraging the potential of tumor-specific molecules carried by small extracellular vesicles (EVs).

**Methods:**

The study utilized both internal and external cohorts of HCC patients and controls. Small EVs were isolated from serum samples, then characterized and validated to confirm their identity. The expression levels of *EV-GULP1* were quantified using quantitative reverse transcription polymerase chain reaction (qRT-PCR).

**Results:**

*EV-GULP1* expression was found to be significantly higher in HCC patients, including those with early-stage disease, when compared to control groups. It demonstrated superior diagnostic accuracy over AFP, achieving an area under the curve (AUC) of 0.919, and was particularly effective in detecting AFP-negative cases. Furthermore, high *EV-GULP1* expression correlated with worse overall and disease-free survival outcomes.

**Conclusion:**

These findings highlight *EV-GULP1* as a highly promising noninvasive biomarker for hepatocellular carcinoma. It offers improved diagnostic accuracy for early detection and better risk stratification for prognosis compared to the current standard, AFP.

## 1 Introduction

Hepatocellular carcinoma (HCC) is the predominant form of primary liver cancer, accounting for approximately 90% of all liver cancer cases. This poses a significant health burden globally. It is the sixth most common cancer worldwide, and the third leading cause of cancer-related deaths globally [1–3]. Moreover, its annual incidence is expected to surpass 1 million new cases by 2025, with a 5-year survival rate of only 18% [4,5]. Major risk factors for HCC include chronic infection with the hepatitis B virus (HBV) or hepatitis C virus (HCV), excessive alcohol consumption, nonalcoholic fatty liver disease, aflatoxin B1 exposure, and smoking [6,7].

Despite significant advances in the understanding of the oncogenic processes of HCC, current surveillance methods primarily rely on abdominal ultrasonography and serum alpha-fetoprotein (AFP) measurements in high-risk populations, such as those with HBV, HCV, or liver cirrhosis (LC) [8,9]. However, abdominal ultrasonography has low sensitivity for early HCC detection, and liver biopsies pose medical challenges owing to the risk of bleeding. Thus, more effective and non-invasive HCC diagnostic methods are urgently required [10,11]. To overcome these limitations, liquid biopsy has emerged as a promising tool, offering the potential for early detection through the analysis of circulating biomarkers [12–14].

GULP PTB domain-containing engulfment adaptor 1 (GULP1), which is the human equivalent of *ced-6* in *Caenorhabditis elegans*, plays a conserved role in phagocytosis [15]. It contributes to cellular processes, such as trogocytosis and engulfment of apoptotic cells, thereby maintaining tissue homeostasis [16]. Given its fundamental role in these processes, GULP1 may influence the development of diseases, such as cancer. GULP1 expression is often diminished in several cancer types, supporting its designation as a tumor suppressor gene [16,17]. However, emerging evidence reveals a contrasting trend in HCC, where GULP1 expression may be elevated and potentially oncogenic. In our earlier work, we not only observed that GULP1 was overexpressed in HCC cells, but also noted that the increased protein level of GULP1 correlated strongly with advanced disease stage and higher recurrence rates [18].

In the present study, we aimed to expand on our previous findings that *GULP1* is not only overexpressed intracellularly and at the transcript level in HCC cells but also secreted into the extracellular environment via small extracellular vesicles (EVs). Based on this observation, we hypothesized that EV-associated GULP1 (*EV-GULP1*) in patient serum may serve as a novel biomarker for hepatocellular carcinoma (HCC), with enhanced diagnostic and prognostic value compared to the conventional marker, alpha-fetoprotein (AFP). Specifically, we investigated whether *EV-GULP1* levels could improve HCC detection, particularly in early-stage and AFP-negative patients, and provide clinically relevant prognostic information.

## 2 Materials and Methods

### 2.1 Analysis of Publicly Available EV Datasets

To identify differentially expressed genes in EVs derived from patients with HCC, we analyzed publicly available RNA-seq data from the GSE199509 dataset (Gene Expression Omnibus, https://www.ncbi.nlm.nih.gov/geo/query/acc.cgi?acc=GSE199509, accessed on 01 January 2025) [19]. This dataset includes the transcriptomic profiles of EVs isolated from patients with LC, early-stage HCC (eHCC), and advanced HCC (aHCC). Hierarchical clustering and differential expression analyses were performed to identify key upregulated genes in HCC-derived EVs.

### 2.2 Patient Recruitment and Clinical Data Collection

To ensure objectivity and reduce institutional bias, initial discovery and diagnostic evaluation were conducted using a discovery cohort composed exclusively of samples from three independent external biobanks: Seoul National University Hospital Biobank (Seoul, Republic of Korea), the Biobank of Keimyung University Dongsan Hospital (Daegu, Republic of Korea), and the Bank of KIRAMS Radiation (Seoul, Republic of Korea). This external discovery cohort included 29 healthy controls (HCs), 29 patients with chronic hepatitis (CH), 25 patients with LC, and 59 patients with HCC (Table 1).

**Table 1:** Clinicopathological profile of patients in the external discovery cohort

Characteristics	HC (n = 29)	CH (n = 29)	LC (n = 25)	HCC (n = 59)
Age (years), mean ± SD	41.4 ± 14.0	40.3 ± 13.4	57.4 ± 9.7	57.3 ± 9.4
Male sex, n (%)	8 (27.6)	16 (55.2)	13 (52.0)	45 (76.3)
Platelet (×10^3^/mL), mean ± SD	243.1 ± 55.6	222.8 ± 108.9	135.8 ± 43.0	164.4 ± 67.5
Albumin (g/L), mean ± SD	4.6 ± 0.3	4.1 ± 0.4	3.8 ± 0.8	4.1 ± 0.4
Bilirubin (mg/dL), mean ± SD	0.7 ± 0.2	0.9 ± 0.4	1.2 ± 0.7	0.7 ± 0.5
INR, mean ± SD		1.1 ± 0.3	1.2 ± 0.3	1.1 ± 0.1
AST (IU/L), mean ± SD	19.7 ± 5.6	53.4 ± 40.9	45.3 ± 56.6	43.9 ± 20.9
ALT (IU/L), mean ± SD	15.8 ± 6.9	69.1 ± 65.5	44.1 ± 72.5	41.1 ± 27.8
AFP (ng/L), mean ± SD	2.7 ± 0.9	36.4 ± 97.2	66.8 ± 305.5	2994.0 ± 10,749.7
Modified UICC stage, n (%)				
I				28 (47.5)
II				20 (33.9)
III				11 (18.6)
IVA				0 (0)
IVB				0 (0)
GULP1 (log_2_) expression	0.2 ± 1.2	1.3 ± 1.1	0.4 ± 1.2	3.5 ± 1.5

Note: HC, healthy control; CH, chronic hepatitis; LC, liver cirrhosis; HCC, hepatocellular carcinoma; SD, standard deviation; INR, international normalized ratio; AST, aspartate aminotransferase; ALT, alanine aminotransferase; AFP, α-fetoprotein; UICC, Union for International Cancer Control.

All experiments were performed in accordance with the Declaration of Helsinki, and the study was approved by the Institutional Review Board of Ajou University Hospital (AJIRB-BMR-KSP-16-365, AJIRB-BMR-SMP-17-189, AJOUIRB-KSP-2019-417, AJOUIRB-EX-2022-389 and AJOUIRB-EX-2024-332). Anonymous serum samples and clinical data were provided by the Ajou Human Bio-Resource Bank; the requirement for informed consent was waived.

For subsequent in-depth validation, an internal cohort was established using samples and clinical data from the Biobank of Ajou University Hospital (Suwon, Republic of Korea). The cohort comprised 29 HCs, 29 patients with CH, 31 patients with LC, and 65 patients with HCC. In contrast to the external cohort, the Ajou cohort was enriched with detailed clinical parameters, enabling extended analysis of the Barcelona Clinic Liver Cancer (BCLC) stage, overall survival (OS), and disease-free survival (DFS), particularly in patients with HCC (Table 2) [20].

**Table 2:** Clinicopathological profile of patients in the internal validation cohort

Characteristics	HC (n = 29)	CH (n = 29)	LC (n = 31)	HCC (n = 65)
Age (years), mean ± SD	34.4 ± 7.8	50.3 ± 0.5	47.2 ± 6.3	54.1 ± 8.6
Male sex, n (%)	3 (10.3)	15 (53.6)	17 (54.8)	48 (73.8)
Etiology, n (%)				
HBV				56 (86.2)
HCV				2 (3.1)
Alcohol				3 (4.6)
Others				0 (0)
Platelet (×10^3^/mL), mean ± SD	68.5 ± 135.9	138.0 ± 39.1	167.3 ± 112.8	168.2 ± 89.7
Albumin (g/L), mean ± SD		4.0 ± 1.4	4.2 ± 0.2	4.2 ± 0.6
Bilirubin (mg/dL), mean ± SD		0.9 ± 0.4	1.0 ± 1.1	1.6 ± 4.1
INR, mean ± SD		0.1 ± 0.3	1.1 ± 0.5	6.2 ± 5.7
AST (IU/L), mean ± SD	16 ± 3.4	92.3 ± 40.5	47.6 ± 19.3	81.8 ± 110.4
ALT (IU/L), mean ± SD	13.4 ± 5.6	148.0 ± 114.2	61.8 ± 43.1	54.1 ± 67.5
AFP (ng/L), mean ± SD	1.8 ± 0.7	19.2 ± 24.8	27.1 ± 34.3	4333.7 ± 14,721.2
Modified UICC stage, n (%)				
I				23 (35.4)
II				14 (21.5)
III				15 (23.1)
IVA				8 (12.3)
IVB				5 (7.7)
BCLC stage, n (%)				
0				22 (33.8)
A				11 (16.9)
B				12 (18.5)
C				20 (30.8)
D				0 (0)
RFS (months), mean ± SD				26.5 ± 24.3
OS (months), mean ± SD				37.1 ± 24.8

Note: HC, healthy controls; CH, chronic hepatitis; LC, liver cirrhosis; HCC, hepatocellular carcinoma; HBV, hepatitis B virus; HCV, hepatitis C virus; AST, aspartate aminotransferase; ALT, alanine aminotransferase; AFP, α-fetoprotein; INR, international normalized ratio; UICC, Union for International Cancer Control; BCLC, Barcelona Clinic Liver Cancer; SD, standard deviation; RFS, recurrence-free survival; OS, overall survival.

The following clinical data were collected from all subjects: age, sex, platelet count, aspartate aminotransferase (AST), alanine aminotransferase (ALT), serum AFP, protein induced by vitamin K absence II (PIVKA-II), etiology of liver disease, serum albumin, total bilirubin, and international normalized ratio (INR). Tumor stage and vascular invasion were assessed using the modified Union for International Cancer Control (mUICC) classification system [21]. Patients without AFP data were excluded from the receiver operating characteristic (ROC) and precision-recall (PR) curve analyses in both the discovery and validation cohorts.

The clinicopathological characteristics of the external and internal cohorts are summarized in Tables 1 and 2, respectively.

### 2.3 Serum Sample Collection and Processing

For serum collection, 5 mL whole blood was drawn from each participant and placed in serum separator tubes (without anticoagulants). Blood samples were left at room temperature for clotting and subsequently centrifuged to remove red blood cells. The serum fraction was transferred into RNA-free Eppendorf tubes and centrifuged again at 2000× *g* for 5 min at 4°C to eliminate residual cellular debris. All processed sera samples were stored at −80°C until further analysis.

### 2.4 Isolation of Serum-Derived Small EVs

Small EVs were isolated from human serum using ExoQuick (Catalog number EXOQ20A-1, System Biosciences, Mountain View, CA, USA) following the manufacturer’s protocol with slight procedural adjustments [22]. In brief, 300 μL of serum was mixed with 72 μL of ExoQuick and incubated at 4°C overnight to facilitate EV precipitation. The samples were subsequently centrifuged at 1500× *g* for 30 min at room temperature. Following the removal of the supernatant, we resuspended it in 100 μL of phosphate-buffered saline (PBS, pH 7.4) and preserved it at −80°C for downstream RNA and protein extraction.

### 2.5 Transmission Electron Microscopy (TEM)

The morphology of the isolated small EVs was visualized by TEM. EVs were fixed with 2% glutaraldehyde and 4% paraformaldehyde, and labeled with a 10-nm gold-conjugated anti-CD63 antibody (Catalog number sc-5275; 1:50; Santa Cruz Biotechnology, Inc., Dallas, TX, USA) before visualization. Images were acquired using a Sigma 500 electron microscope (Carl Zeiss, Oberkochen, Germany).

### 2.6 Nanoparticle Tracking Analysis (NTA)

The size distribution and concentration of serum-derived small EVs were determined by NTA using the NanoSight NS300 instrument (Malvern Panalytical, UK) equipped with a 405-nm laser. Three 60-s videos were recorded per sample, and the data were analyzed using NTA software (version 3.0).

### 2.7 Western Blot Analysis

For Western blotting, serum-derived small EVs and Hep3B total cell lysates were lysed in RIPA buffer (100 μL; Thermo Scientific, Waltham, MA, USA) and incubated on ice for 10 min. The Hep3B cell line was obtained from the Korean Cell Line Bank (KCLB, Seoul, Republic of Korea) and authenticated by short tandem repeat (STR) profiling. Mycoplasma contamination was routinely tested using PCR-based methods and found to be negative.

Total protein concentrations were measured using the bicinchoninic acid (BCA) assay (Catalog number 23225; Thermo Scientific). Equal amounts of protein (10 μg) were separated on 4%–20% Mini-PROTEAN TGX™ gels (Bio-Rad Laboratories, Hercules, CA, USA) and transferred onto polyvinylidene difluoride (PVDF) membranes (Amersham; GE Healthcare, Munich, Germany).

Membranes were blocked with 5% nonfat milk in TBS-T and incubated with the following primary antibodies: rabbit anti-CD9 (Catalog number ab92726; Abcam, Cambridge, UK; 1:2000), rabbit anti-CD63 (Catalog number ab134045; Abcam; 1:1000), and mouse anti-BiP/GRP78 (Catalog number 610979; BD Biosciences, San Jose, CA, USA; 1:1000). After washing, membranes were probed with HRP-conjugated secondary antibodies: anti-rabbit (Catalog number BR170-6515; Bio-Rad Laboratories Inc.; 1:3000) or anti-mouse (Catalog number BR170-6516; Bio-Rad Laboratories Inc.; 1:5000).

Full, uncropped, and unedited Western blot images are provided in Supplementary Fig. S1.

### 2.8 RNA Extraction and Quantitative Reverse Transcription Polymerase Chain Reaction (qRT-PCR) Analysis

Total RNA was isolated from serum and EV samples using TRIzol-LS reagent (Catalog number 10296028; Invitrogen, Carlsbad, CA, USA) following the manufacturer’s instructions. Reverse transcription was performed with the PrimeScript™ RT Master Mix (Catalog number RR036A; TaKaRa Bio, Otsu, Japan) for tissue- and cell-derived RNA, and with the miScript II RT Kit (Catalog number 218161; QIAGEN, Hilden, Germany) or the Biotechrabbit cDNA Synthesis Kit (Catalog number BR0400403; Biotechrabbit, Hennigsdorf, Germany) for serum EV-derived RNA. qRT-PCR was carried out using the AmfiSure qGreen Q-PCR Master Mix (Catalog number Q5602; GenDEPOT, Barker, TX, USA) on an Applied Biosystems 7300 Real-Time PCR System (Applied Biosystems, Foster City, CA, USA) or a Bio-Rad CFX Connect Real-Time PCR Detection System (Bio-Rad Laboratories).

The following primers were used: *GULP1* (forward: 5^′^-AACGGGACCTGTTTGGAGCA-3^′^, reverse: 5^′^-ACCCCTCCTGCATCTCATCT-3^′^) and *HMBS* (reference gene: forward: 5^′^-GGAGGGCAGAAGGAAGAAAACAG-3^′^, reverse: 5^′^-CACTGTCCGTCTGTATGCGAG-3^′^).

To ensure consistency across different batches of EV analysis, we applied systematic quality control measures. *HMBS* was selected as the internal reference gene based on its validated expression stability in our previous studies [22–24]. All qRT-PCR reactions were performed in technical triplicate, and pooled healthy control samples were included in each run to monitor inter-batch variability. For qRT-PCR analysis of MIHA cells, the cells were provided by Dr. Roy-Chowdhury (Albert Einstein College of Medicine, Bronx, NY, USA), authenticated by STR profiling, and confirmed to be free of mycoplasma contamination.

### 2.9 Statistical Analysis

Data are presented as mean values ± standard deviation of three experiments. The statistical significance of the differences between the two groups was assessed using the paired Student’s *t*-test or unpaired Welch’s *t*-test using GraphPad Prism (version 10.0; GraphPad Software, San Diego, CA, USA). One-way analysis of variance with Tukey’s post-hoc test was conducted for multi-group comparison. Violin plots were generated using GraphPad Prism to visualize the distribution of *EV-GULP1* expression levels. All experiments were performed at least three times. Statistical significance was set at *p < 0.05. For the diagnostic evaluation of *EV-GULP1*, we performed ROC curve analysis on the ddCt (log_2_) values to determine the optimal cutoff. We used Youden’s Index, a widely accepted statistical method that identifies the optimal threshold by maximizing the sum of sensitivity and specificity. The analysis was conducted using MedCalc statistical software (MedCalc Software, version 22.018; Ostend, Belgium), and the optimal cutoff value for *EV-GULP1* was determined to be 1.35. For the conventional biomarker AFP, we used the widely accepted clinical cutoff value of 20 ng/mL.

## 3 Results

### 3.1 Serum-Derived Small EV-GULP1 Expression in HCC

**Figure 1:**
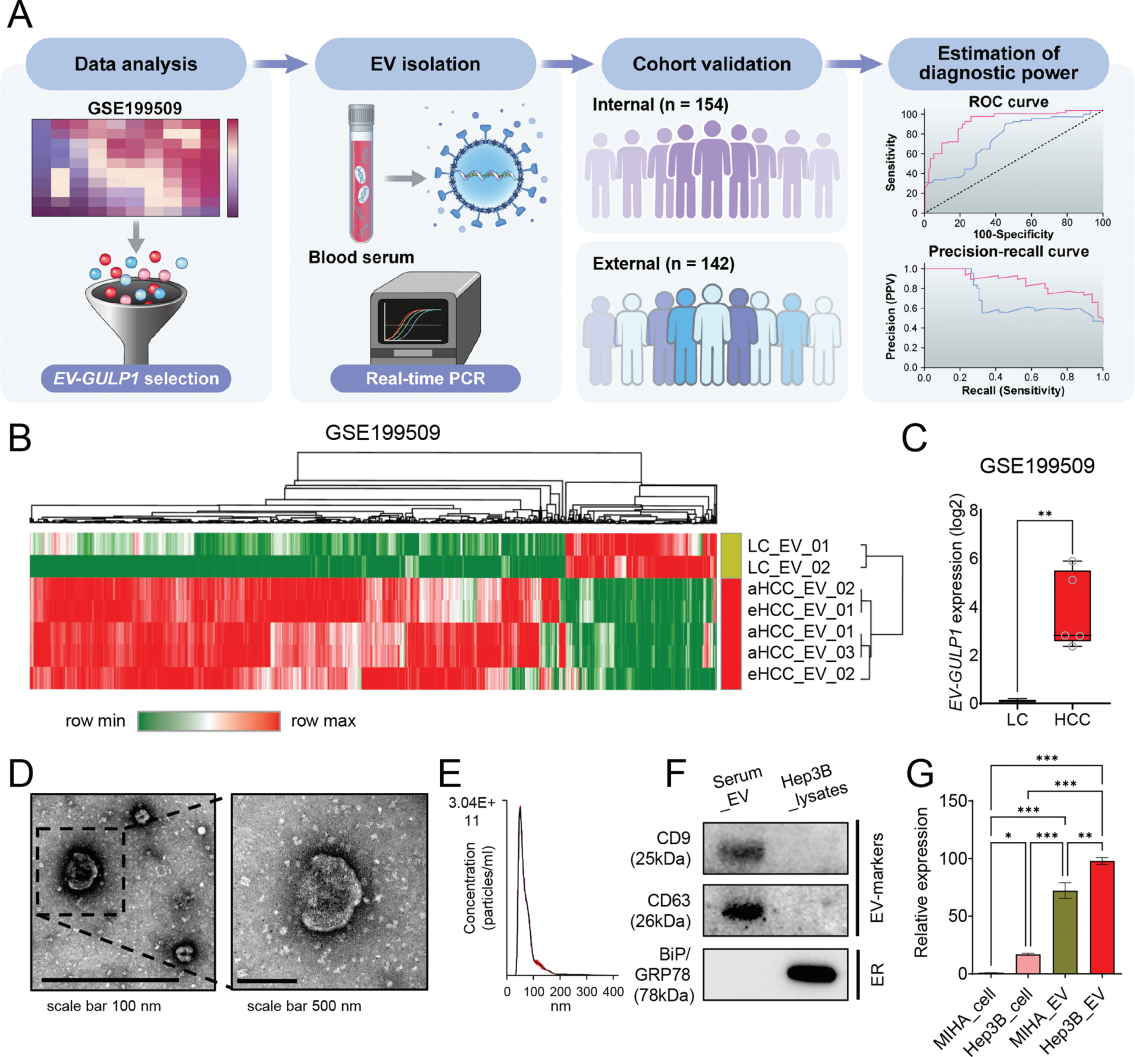
Serum-derived *EV-GULP1* expression in patients with HCC. (**A**) Flow summary of the research process for small extracellular vesicle-encapsulated *GULP1* (*EV-GULP1*) analysis in HCC. (**B**) Heatmap and hierarchical clustering analysis of RNA-seq data from the GSE199509 dataset comparing EVs derived from patients with liver cirrhosis (LC), early-stage HCC (eHCC), and advanced HCC (aHCC). *GULP1* was identified as one of the most upregulated transcripts in HCC-derived EVs. (**C**) Box plot showing significantly elevated expression of *EV-GULP1* in patients with HCC compared to patients with LC (*p* < 0.01). (**D**) Characterization of small EVs by transmission electron microscopy (TEM) confirmed the presence of vesicles with typical morphology and size. (**E**) Nanoparticle tracking analysis (NTA) revealed particle size distribution consistent with small EVs. (**F**) Western blot analysis of serum-derived small EVs and Hep3B cell lysates showed the presence of EV markers CD9 and CD63, whereas the negative and endoplasmic reticulum (ER) marker BiP/GRP78 was detected only in the cell lysate, confirming the purity of the isolated EVs. (**G**) Quantitative reverse transcription polymerase chain reaction analysis comparing *GULP1* expression in MIHA cells, Hep3B cells, MIHA-derived EVs, and Hep3B-derived EVs, showing significantly increased expression of *GULP1* in Hep3B EVs compared to MIHA EVs. *p < 0.05, **p < 0.01, and ***p < 0.001

To investigate alterations in EV-encapsulated gene expression in HCC, we analyzed RNA-seq data from the GSE199509 dataset, comparing LC, eHCC, and aHCC cases, and validated our findings across discovery (n = 142) and validation (n = 154) cohorts. This comprehensive approach included serum-derived small EV isolation, qRT-PCR-based gene expression evaluation, and subsequent diagnostic and prognostic assessments. A summary of the workflow is presented in Fig. 1A.

Initially, we analyzed RNA-seq data from the GSE199509 dataset to identify differentially expressed genes in EVs from patients with HCC, including both eHCC and aHCC, compared with LC controls. Among the 39,276 detected genes, we initially selected genes with log_2_ (transcripts per million [TPM] + 1) values ≥ 0.1 in HCC group, based on their mean expression in LC, to define genes that are actively expressed in HCC-derived EVs. We then applied Welch’s *t*-test to compare expression between LC and HCC groups, selecting 554 genes with **p < 0.01 and fold change ≥ |3| as differentially expressed candidate genes.

Hierarchical clustering and heatmap analysis of the GSE199509 dataset showed that 554 genes were aberrantly regulated in HCC-derived EVs, which displayed a distinctive gene expression pattern that clearly distinguished the LC from HCC group (Fig. 1B). These results suggest that numerous EV-loaded genes may drive the development of HCC or may be altered in response to hepatocarcinogenesis. Building on our previous finding that serum GULP1 protein levels exhibit diagnostic potential in patients with HCC, we further determined whether *EV-GULP1* retained similar clinical utility [18].

*EV-GULP1* expression was significantly higher in both eHCC and aHCC than in LC, suggesting its potential role as a biomarker of HCC progression (Fig. 1C). To validate the presence and morphology of serum-derived EVs, we employed TEM. Microscopic images revealed vesicles with typical lipid bilayer membranes, and a higher-magnification view further confirmed their spherical morphology, consistent with that of small EVs (Fig. 1D). NTA was conducted to assess the size distribution and concentration of the isolated EVs, which showed that most vesicles were within the expected diameter range, confirming successful isolation (Fig. 1E). Western blot analysis verified the purity of the isolated EVs and the expression levels of *GULP1* in serum-derived EVs and Hep3B_lysates. As shown in the serum_EV lane in Fig. 1F, the EV markers CD9 and CD63 were readily detected, whereas the non-EV marker BiP/GRP78 was absent, confirming the successful isolation and purity of the EV fraction. In contrast, Hep3B_lysates exhibited minimal expression of EV markers, but high BiP/GRP78 levels, indicating their intracellular origin and distinguishing them from EVs (Fig. 1F; Hep3B lysate lane).

To further characterize *GULP1* expression in normal and HCC tissues, we measured its levels in MIHA (normal hepatocytes) and Hep3B (liver cancer) cells, as well as in their respective EVs (Fig. 1G). *GULP1* expression was significantly higher in Hep3B cells than that in MIHA cells, suggesting its association with HCC. *GULP1* levels were also elevated in Hep3B-derived EVs compared to those in MIHA-derived EVs, suggesting that EV-mediated transfer of *GULP1* may contribute to HCC pathogenesis.

### 3.2 HCC Diagnostic Performance of EV-GULP1 Compared to AFP

**Figure 2:**
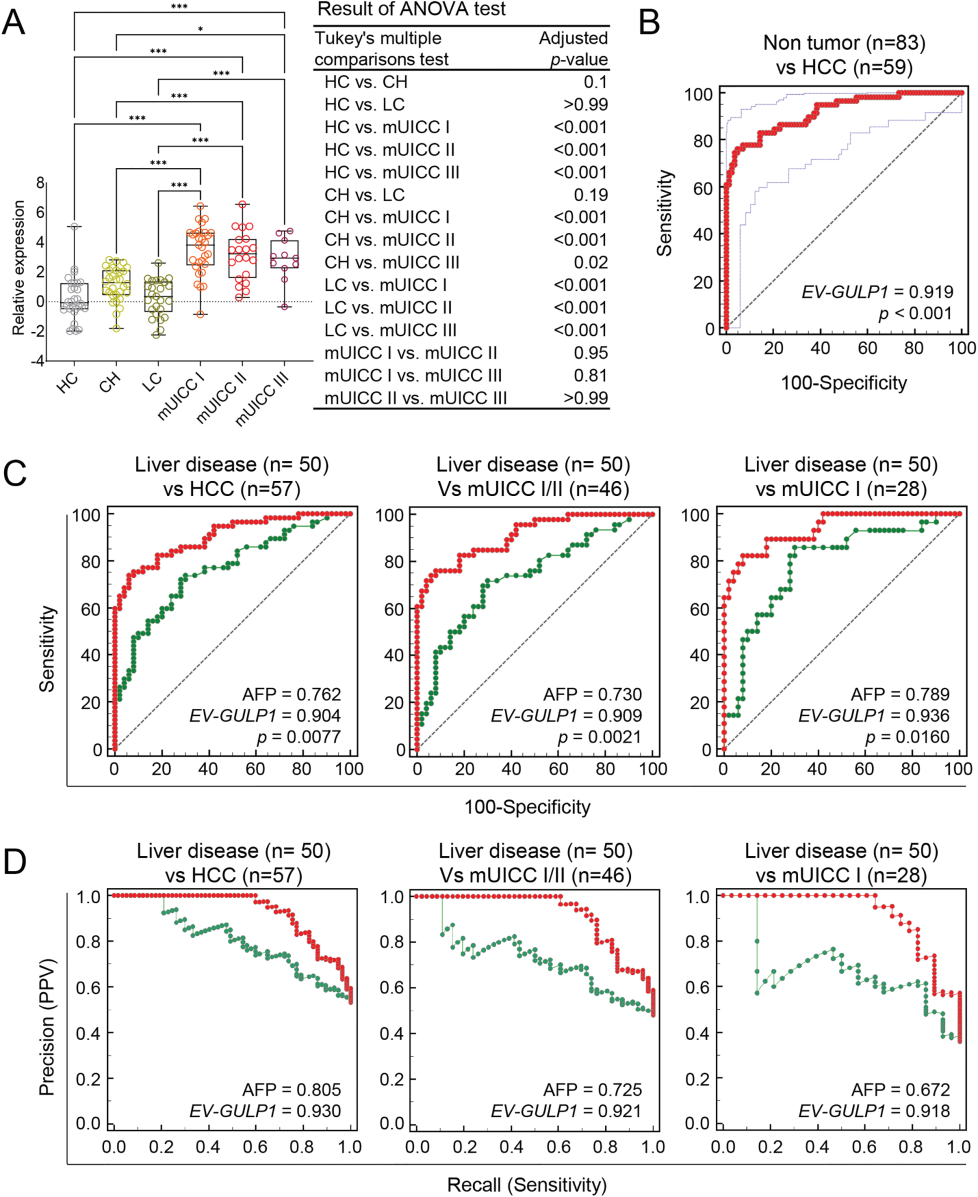
Diagnostic performance of *EV-GULP1* compared to AFP for HCC detection. (**A**) Serum-derived *EV-GULP1* expression levels in an external discovery cohort comprising healthy controls (HC), patients with chronic hepatitis (CH), patients with liver cirrhosis (LC), and those with HCC stratified by mUICC stages I, II, and III. *EV-GULP1* levels were significantly increased in patients with HCC compared to healthy controls. (**B**) ROC curve analysis assessing the ability of *EV-GULP1* to distinguish HCC from non-HCC conditions in the external discovery cohort, demonstrating high diagnostic accuracy. (**C**) ROC curve analysis comparing the performance of *EV-GULP1* and AFP in differentiating HCC from patients with liver disease (CH and LC) in the external cohort. Patients with missing AFP values were excluded from the analysis. (**D**) PR curve analysis confirming that *EV-GULP1* maintains superior classification performance compared to AFP in distinguishing HCC from liver disease in the external discovery cohort. Individuals lacking AFP data were not included in the analysis. Data are presented as means ± standard errors of the mean. *p < 0.05 and ***p < 0.001. ANOVA, analysis of variance; mUICC, modified Union for International Cancer Control; PPV, positive predictive value

The diagnostic performance of *EV-GULP1* was further examined in an independent discovery cohort using sera samples obtained from the Seoul National University Hospital Biobank (Seoul, Republic of Korea), Biobank of Keimyung University Dongsan Hospital (Daegu, Republic of Korea), and the Bank of KIRAMS Radiation (Seoul, Republic of Korea). The discovery cohort included 29 HCs, 29 patients with CH, 25 patients with LC, and 59 patients with HCC, further stratified into mUICC stages I (n = 28), II (n = 20), and III (n = 11) (Table 1). To determine the relationship between *EV-GULP1* expression and disease stage, we first examined its levels across different disease groups (Fig. 2A). *EV-GULP1* expression was significantly higher in patients with mUICC I than in those with non-tumor conditions, and these elevated levels were maintained consistently in stages II and III rather than showing a gradual increase. This pattern suggests that *EV-GULP1* undergoes a marked increase upon malignant transformation and remains elevated as the disease progresses, reinforcing its strong association with HCC rather than with incremental disease advancement. The ability of *EV-GULP1* to differentiate HCC from non-tumor conditions was further analyzed using ROC curve analysis (Fig. 2B). The results demonstrated high diagnostic accuracy, with an AUC of 0.919 (***p < 0.001) for distinguishing HCC from non-tumor conditions, supporting its potential as a reliable biomarker.

A key challenge in HCC diagnosis is distinguishing malignant from non-malignant liver conditions, as traditional biomarkers, such as AFP, often yield positive results in patients with chronic liver disease, despite their intended use for cancer detection. Although AFP levels commonly remain negative in HCs, they frequently increase in patients with CH and LC, leading to false-positive results in non-malignant conditions [25,26]. Considering this limitation, assessing the ability of *EV-GULP1* to differentiate HCC from the underlying liver disease is crucial for biomarker validation. Therefore, we performed an additional set of ROC and PR curve analyses within the external discovery cohort to evaluate *EV-GULP1*’s performance in distinguishing patients with HCC from those with liver disease.

Consequently, we conducted an independent analysis using an internal cohort to assess the diagnostic performance of *EV-GULP1* in distinguishing HCC from non-malignant liver diseases including CH and LC. ROC analysis confirmed that *EV-GULP1* maintained a strong ability to differentiate non-tumor conditions from HCC, achieving an AUC of 0.904 (*p* = 0.0077), whereas AFP showed a lower AUC of 0.762 (Fig. 2C, left panel). Additional analyses further reinforced its high diagnostic accuracy, with AUCs of 0.909 and 0.936 in different comparisons (*p* = 0.0021 and 0.016, respectively), which were superior to those of AFP (AUCs of 0.730 and 0.789, respectively) (Fig. 2C, middle and right panels, respectively). PR curve analysis, which accounted for class imbalance and provided a more clinically relevant evaluation, also highlighted the superior classification performance of *EV-GULP1*, with AUCs of 0.930, 0.921, and 0.918, whereas AFP exhibited lower AUCs of 0.805, 0.725, and 0.672, respectively (*p < 0.05) (Fig. 2D).

Furthermore, ROC curve analysis using the external cohort demonstrated that *EV-GULP1* maintained high diagnostic accuracy in distinguishing HCC from non-tumor conditions, including HC and patients with CH or LC. *EV-GULP1* achieved AUCs of 0.920, 0.925, and 0.948 across different comparisons—non-tumor vs. HCC, non-tumor vs. mUICC I/II, and non-tumor vs. mUICC I, respectively (*p* = 0.0183, 0.0054, and 0.0448, respectively) (Supplementary Fig. S2A). These values were consistently higher than those of AFP (AUCs of 0.815, 0.788, and 0.843, respectively), supporting the superior discriminative power of *EV-GULP1* even in early-stage HCC. PR curve analysis using the external cohort also revealed that *EV-GULP1* significantly outperformed AFP in terms of precision and recall, with AUCs of 0.917, 0.907, and 0.907, respectively, compared with 0.790, 0.707, and 0.664 for AFP, respectively (Supplementary Fig. S2B).

These findings underscore the ability of *EV-GULP1* to accurately distinguish patients with HCC from healthy individuals or patients with chronic liver disease, addressing the key limitation of conventional biomarkers that often yield false positives in non-malignant conditions. Its capacity to maintain high specificity while preserving superior sensitivity for HCC detection highlights its strong clinical potential as a reliable and noninvasive biomarker, particularly in high-risk populations with chronic liver disease.

### 3.3 Additional Validation of EV-GULP1 as a Diagnostic Biomarker for HCC

**Figure 3:**
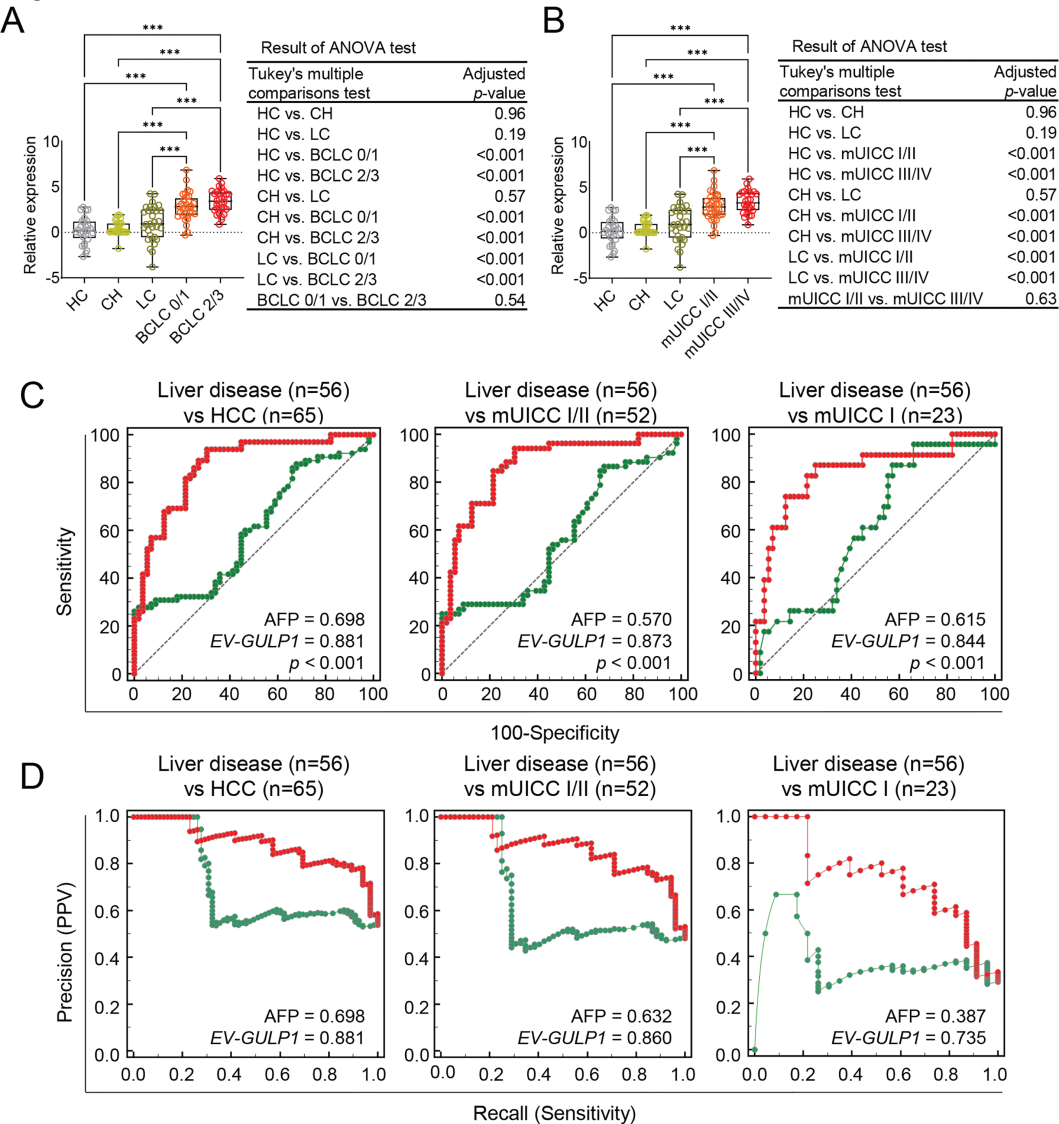
Internal validation of *EV-GULP1* diagnostic performance in an independent cohort. (**A**) Serum-derived *EV-GULP1* expression levels according to Barcelona Clinic Liver Cancer (BCLC) stages, showing significantly higher expression levels in patients with advanced-stage HCC. (**B**) Serum-derived *EV-GULP1* expression levels stratified by modified Union for International Cancer Control (mUICC) stages, demonstrating a significant increase in patients with HCC compared to healthy controls. Data are presented as means ± standard errors of the mean. (**C**) ROC curve analysis evaluating the performance of *EV-GULP1* and AFP in distinguishing HCC from patients with liver disease (chronic hepatitis and liver cirrhosis). Patients with missing AFP values were excluded from the analysis. (**D**) PR curve analysis confirming the superior precision and recall of *EV-GULP1* over AFP in differentiating HCC from liver disease groups. Cases without available AFP values were excluded from the study. ***p < 0.001. ANOVA, analysis of variance; mUICC, modified Union for International Cancer Control; PPV, positive predictive value

To further evaluate the clinical relevance of *EV-GULP1* expression across different HCC stages, we analyzed its levels using the BCLC and mUICC classification systems in our internal validation cohort (Table 2). The BCLC classification showed a stepwise increase in *EV-GULP1* expression from BCLC stage 0 (very early stage) to stage C (advanced HCC), with a statistically significant difference between early and advanced disease stages (Fig. 3A). Similarly, the mUICC classification confirmed a progressive increase in *EV-GULP1* levels as the severity of HCC increased (Fig. 3B). Thus, *EV-GULP1* may be strongly associated with HCC progression and may serve as a valuable noninvasive biomarker for both early detection and disease stratification. Notably, to further evaluate its clinical applicability, we assessed the influence of demographic and etiological variables. Our analysis revealed that *EV-GULP1* expression levels were not significantly affected by patient age, sex, or the etiology of their liver cancer (HBV, HCV, or alcohol), highlighting its potential as a universally robust biomarker (Supplementary Fig. S3A–C).

Additionally, we assessed the ability of *EV-GULP1* to differentiate HCC from other liver diseases (CH and LC) using ROC and PR analyses. *EV-GULP1* demonstrated superior accuracy, with AUC values of 0.881, 0.873, and 0.844 for diagnosing all stages of HCC, mUICC stage I or II, and mUICC stage I, respectively, compared to AFP AUCs of 0.698, 0.570, and 0.615, respectively (Fig. 3C). Similarly, PR analysis confirmed the superior diagnostic performance of *EV-GULP1*, with AUCs of 0.881, 0.860, and 0.735, compared to AFP, which showed markedly lower AUCs of 0.698, 0.632, and 0.387, respectively (Fig. 3D).

To evaluate the diagnostic efficacy of serum *EV-GULP1* compared to AFP in distinguishing HCC from non-tumor conditions, we performed ROC and PR curve analyses. ROC curve analysis demonstrated that *EV-GULP1* exhibited significantly higher diagnostic accuracy than AFP across various disease stages. Specifically, for distinguishing non-tumor conditions (HC, CH, and LC) from HCC, *EV-GULP1* achieved an AUC of 0.891, significantly outperforming AFP (AUC = 0.718, ***p < 0.001) (Supplementary Fig. S4A, left panel). This superior performance was also observed for early-stage HCC, as indicated by the mUICC stage I and II subgroup analysis, where *EV-GULP1* exhibited an AUC of 0.893 compared to 0.691 for AFP (***p = 0.001) (Supplementary Fig. S4A, middle panel). In mUICC stage I, *EV-GULP1* maintained a high AUC of 0.866, whereas AFP showed poor performance with an AUC of 0.540 (***p < 0.001) (Supplementary Fig. S4A, right panel). Thus, *EV-GULP1* demonstrated greater sensitivity and specificity than AFP for detecting early-stage HCC.

To validate these findings further, we conducted PR curve analysis. The results of the PR analysis consistently demonstrated that *EV-GULP1* maintained superior precision and recall compared to AFP. Across all HCC stages, *EV-GULP1* had an AUC of 0.862, outperforming AFP (AUC = 0.690) (Supplementary Fig. S4B, left panel). In mUICC stages I and II, the advantage of *EV-GULP1* was evident, with an AUC of 0.842, compared to 0.623 for AFP (Supplementary Fig. S4B, middle panel). Similarly, when distinguishing non-tumor conditions from mUICC stage I HCC, *EV-GULP1* retained an AUC of 0.713, whereas AFP exhibited a significantly lower AUC of 0.250, further highlighting its limited sensitivity in early-stage detection (Supplementary Fig. S4B, right panel). Taken together, these results strongly suggest that *EV-GULP1* is a highly promising biomarker with excellent potential for early detection of HCC, especially for distinguishing early-stage cancer from non-malignant liver conditions.

### 3.4 EV-GULP1 Positivity in Different Disease Groups and Its Comparison with AFP

**Figure 4:**
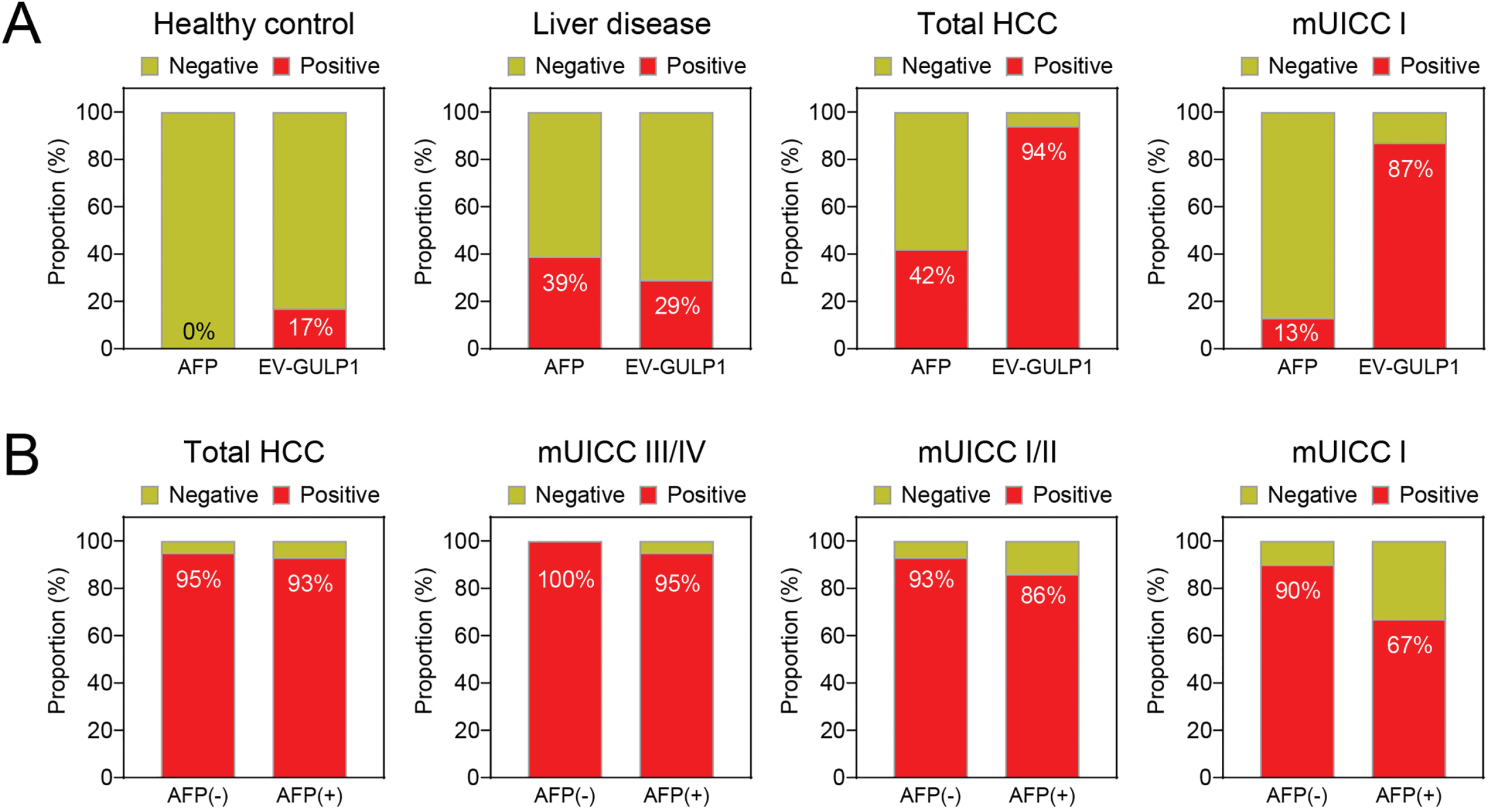
*EV-GULP1* positivity in different disease groups and comparison with AFP. (**A**) Comparison of *EV-GULP1* and AFP positivity rates across healthy controls, liver disease groups (chronic hepatitis and liver cirrhosis), and different HCC stages. *EV-GULP1* exhibited significantly higher positivity rates than AFP in HCC, particularly in early-stage disease. (**B**) *EV-GULP1* positivity rates in AFP-negative (−) and AFP-positive (+) patients with HCC, showing consistently high detection rates in both subgroups, reinforcing its diagnostic utility regardless of AFP status. mUICC, modified Union for International Cancer Control

To compare the detection rates of *EV-GULP1* and AFP across different disease conditions, we analyzed their positivity rates in HCs, patients with liver disease, and patients at various stages of HCC (Fig. 4A). For this analysis, *EV-GULP1* positivity was defined as a ddCt (log_2_) value of 1.35 or greater, while AFP positivity was defined as a value exceeding 20 ng/mL. Among the HCs, AFP showed no positivity (0%), whereas *EV-GULP1* was detected in 17% of the cases, suggesting a slightly higher baseline presence in non-HCC conditions (Fig. 4A, first panel). In patients with liver disease, including CH and LC, AFP had a positivity rate of 39%, whereas *EV-GULP1* was detected in 29% of the cases (Fig. 4A, second panel). The lower positivity rate of *EV-GULP1* in liver disease compared to that of AFP suggests higher specificity, potentially reducing false positives from non-malignant liver conditions.

AFP positivity increased to 42% in patients with HCC across all stages; however, *EV-GULP1* demonstrated a significantly higher positivity rate of 94%, indicating superior sensitivity for HCC detection (Fig. 4A, third panel). In patients with mUICC stage I, AFP was positive in only 13% of cases, whereas *EV-GULP1* exhibited a considerably higher positivity rate of 87%, further supporting its strong potential for detecting early-stage HCC (Fig. 4A, fourth panel). Although AFP retains its diagnostic utility, *EV-GULP1* provides superior sensitivity, particularly in early-stage HCC, making it a reliable biomarker for liver cancer detection.

We further analyzed the positivity rates of *EV-GULP1* in AFP-negative (AFP (−)) and AFP-positive (AFP (+)) patients with HCC across different stages. *EV-GULP1* consistently exhibited high positivity rates in both AFP (−) and AFP (+) patients, with minimal variation between the two groups. In AFP (−) HCC cases, where AFP typically fails as a diagnostic marker, *EV-GULP1* remained highly detectable, exceeding 90% positivity across all stages, confirming its high sensitivity, independent of AFP status (Fig. 4B). Specifically, *EV-GULP1* was detected in 93% of AFP (−) cases in mUICC stage I/II, whereas its detection rate was 100% in advanced HCC cases of mUICC stage III/IV (Fig. 4B, second to fourth panels). Even in AFP (+) cases, *EV-GULP1* positivity remained consistently high, with 86% detection in mUICC stage I/II and 95% detection in advanced HCC cases of mUICC stage III/IV, emphasizing its robustness as a diagnostic tool (Fig. 4B). These findings clearly demonstrate that *EV-GULP1* provides reliable diagnostic performance regardless of AFP status.

### 3.5 Prognostic Significance of EV-GULP1 in HCC Survival Outcomes

**Figure 5:**
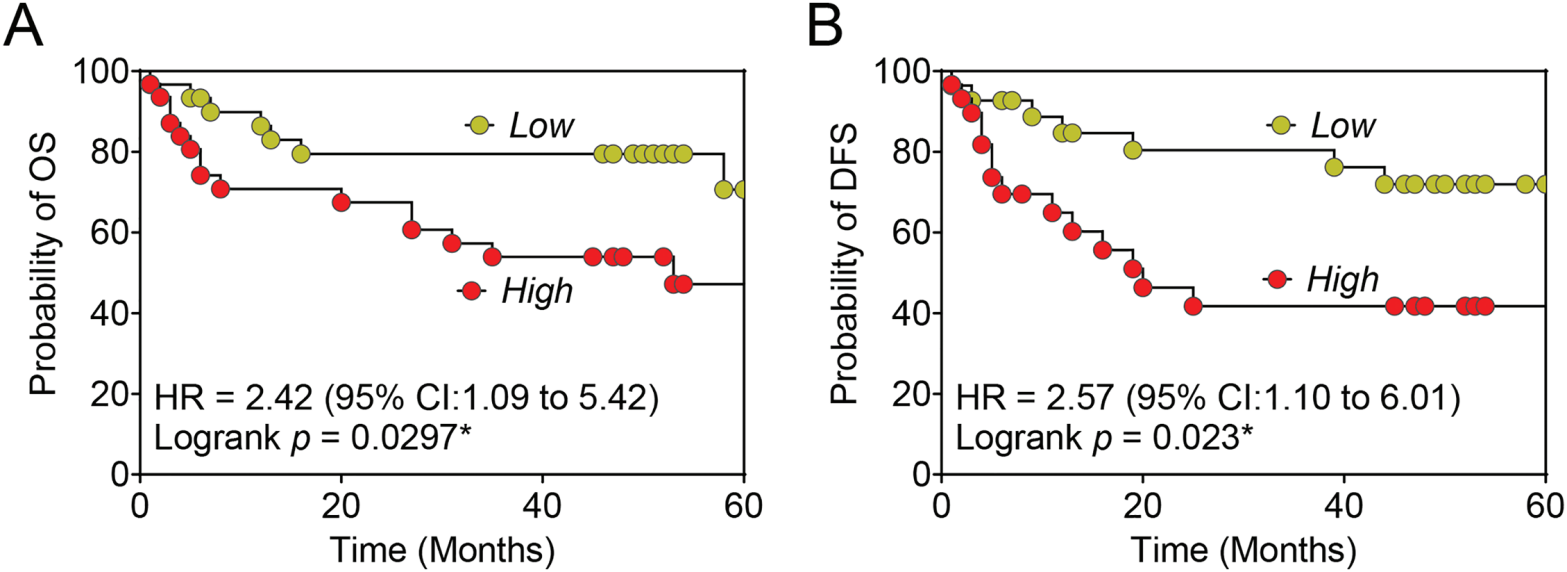
Prognostic significance of *EV-GULP1* in HCC survival outcomes. (**A**) Kaplan–Meier survival curves for overall survival (OS) stratified by high and low *EV-GULP1* expression groups. Patients with high *EV-GULP1* expression levels exhibited significantly worse OS than those with low *EV-GULP1* expression levels (*p* = 0.0297). (**B**) Kaplan–Meier survival curves for disease-free survival (DFS), showing a significant association between high *EV-GULP1* expression and shorter DFS (*p* = 0.023). HR, hazard ratio; CI, confidence interval. *p < 0.05

To assess the prognostic significance of *EV-GULP1* in HCC, we performed Kaplan–Meier survival analyses for OS and DFS based on *EV-GULP1* expression levels. Patients with high *EV-GULP1* expression levels exhibited significantly worse OS than those with low *EV-GULP1* expression levels (hazard ratio [HR] = 2.42, 95% confidence interval [CI]: 1.09–5.42, *p* = 0.0297), indicating that elevated *EV-GULP1* levels were associated with more aggressive disease and reduced survival rates (Fig. 5A). Similarly, patients with high *EV-GULP1* expression levels had a significantly shorter DFS than those with low *EV-GULP1* expression levels (HR = 2.57, 95% CI: 1.10–6.01, *p* = 0.023), suggesting that higher *EV-GULP1* levels are correlated with a greater likelihood of early disease recurrence or progression following treatment (Fig. 5B). These findings highlight *EV-GULP1* as a strong prognostic biomarker for HCC that can predict both reduced OS and shorter DFS. The significant association between high *EV-GULP1* expression levels and poor survival outcomes supports its potential role in HCC progression and underscores its clinical relevance for patient risk stratification. *EV-GULP1* may primarily indicate tumor aggressiveness and post-treatment recurrence, further emphasizing its utility as a prognostic biomarker for HCC management.

## 4 Discussion

Various guidelines have been established for primary liver cancer; however, recurrence and progression remain formidable challenges despite diverse therapeutic strategies, including surgical resection, ablation, and liver transplantation [27–29]. Although AFP has historically served as a conventional biomarker, its limited sensitivity for early-stage disease underscores the urgent need for novel indicators [30,31].

Recent developments in liquid biopsy, particularly EV analysis, have revealed promising avenues for overcoming this limitation [32–34]. Small EVs are abundantly released by tumor cells and encapsulate proteins, DNA, and various RNA species that mirror the molecular characteristics of their parent cells [35–37]. In the context of HCC, these findings offer a potent complement to standard surveillance methods as they allow minimally invasive, repeatable sampling that may detect subtle molecular signals preceding overt tumor recurrence.

Our previous study has reported that GULP1, initially recognized as a tumor suppressor in other malignancies, functions paradoxically as an oncogene in HCC [18]. Detailed mechanistic analyses revealed that GULP1 augments Wnt/β-catenin signaling—partly mediated via ARF6 activation—and drives epithelial–mesenchymal transition, thereby fostering metastatic dissemination [18]. Despite these mechanistic insights, the prior protein-based or ELISA-based measurement of GULP1 alone faced challenges, including relatively modest area under the curve values in some cohorts. Although serum GULP1 showed promise by outperforming traditional biomarkers such as AFP—particularly in early-stage HCC—its diagnostic utility was still not on par with more established markers, a limitation that other investigators have also pointed out [38].

In contrast, our recent findings on *EV-GULP1* suggest a more tumor-specific and robust approach that may overcome many of these obstacles. Importantly, HCC cells secrete *GULP1* through small EVs, implying that serum-derived *EV-GULP1* levels could serve as a powerful surrogate marker of tumor activity. This aligns with recent research results indicating that EV cargo often reflects the aggressiveness of an underlying disease [39,40]. Our data further reinforce the clinical significance of *GULP1* in that it outperforms AFP in detecting early-stage and AFP-negative tumors, offering higher specificity for HCC than for CH or LC.

However, this study had some limitations. First, our choice of a polymer-based precipitation method (e.g., ExoQuick) for EV isolation warrants discussion. While this approach offers key advantages in scalability, speed, and suitability for the small sample volumes often encountered in clinical biobanks, its main drawback is the potential co-isolation of non-vesicular proteins and macromolecules, which could affect sample purity. Alternative methods, such as ultracentrifugation—often considered the gold standard for high purity—or size-exclusion chromatography, were impractical for this study due to their requirements for larger sample volumes and more intensive labor. Given these constraints, the polymer-based method was the most feasible option, and its efficacy was supported by our preliminary validation experiments. Additionally, while the precise mechanism for GULP1 enrichment in EVs is unknown, we hypothesize it is driven by GULP1’s interaction with the ARF6-syntenin-ALIX exosome biogenesis pathway, a process likely enhanced by tumor-related stress like hypoxia, based on the previous studies [41,42]. Nonetheless, GULP1 expression may also fluctuate in response to LC, underlying inflammation, and coexisting treatments. Therefore, larger-scale validation studies are warranted to refine the cutoff values and strengthen the predictive accuracy of *EV-GULP1*. Combining GULP1 measurements with other extracellular biomarkers or novel liquid biopsy assays may enhance the prognosis and guide personalized therapeutics. Specifically, integrating GULP1 with advanced EV RNA panels could bolster the early detection of microscopic diseases, while simultaneous monitoring of circulating tumor DNA might offer insights into clonal evolution or emerging resistance. Overall, our findings position GULP1 at the intersection of oncogenic signaling and clinically actionable biomarker development. By demonstrating its pivotal role in HCC progression and its detectability in serum-derived EVs, this study proposes *EV-GULP1* as both a practical clinical tool and a potential therapeutic target.

## 5 Conclusion

In summary, our findings support *EV-GULP1* as a promising non-invasive biomarker for HCC. Nonetheless, the retrospective nature of our study involving only Korean cohorts necessitates broader geographic and ethnic validation. Although the sample size was sufficient to meet the primary endpoints, it was limited in-depth subgroup and longitudinal analyses. EVs were isolated via polymer-based precipitation, an established approach that may still contain trace non-vesicular components, underscoring the need to integrate orthogonal purification methods. In future research, harmonizing qRT-PCR cut-off values and confirming these results in prospective, multi-center trials will be essential to fully establish the clinical utility of *EV-GULP1*.

## Supplementary Materials



## Data Availability

The datasets used during the current study are available from the corresponding authors upon reasonable request.

## Supplementary Materials

The supplementary material is available online at https://www.techscience.com/doi/10.32604/or.2025.070392/s1.

## Abbreviations

AFP: Alpha-fetoprotein
ALT: Alanine aminotransferase
AST: Aspartate aminotransferase
AUC: Area under the curve
BCLC: Barcelona Clinic Liver Cancer
CH: Chronic hepatitis
CI: Confidence interval
DFS: Disease-free survival
DNA: Deoxyribonucleic acid
eHCC: Early-stage hepatocellular carcinoma
ELISA: Enzyme-linked immunosorbent assay
ER: Endoplasmic reticulum
EV: Extracellular vesicle
EV-GULP1: Extracellular vesicle-encapsulated GULP1
GULP1: GULP PTB domain-containing engulfment adaptor 1
HBV: Hepatitis B virus
HCC: Hepatocellular carcinoma
HCV: Hepatitis C virus
HC: Healthy control
HR: Hazard ratio
INR: International normalized ratio
LC: Liver cirrhosis
mUICC: Modified Union for International Cancer Control
NTA: Nanoparticle tracking analysis
OS: Overall survival
PIVKA-II: Protein induced by vitamin K absence II
PR: Precision-recall
qRT-PCR: Quantitative reverse transcription polymerase chain reaction
RNA: Ribonucleic acid
RNA-seq: RNA sequencing
ROC: Receiver operating characteristic
TEM: Transmission electron microscopy
TPM: Transcripts per million
