# Supplementary material for: Serum Extracellular Vesicle-Associated GULP1 Is a Key Indicator of Hepatocellular Carcinoma
Source: Oncol Res. 2026 Jan 19;34(2):13. doi: 10.32604/or.2025.070392 (PMC12848681; doi:10.32604/or.2025.070392)
Supplement: Supplementary file 1 [file OncolRes-34-70392-s001.docx]

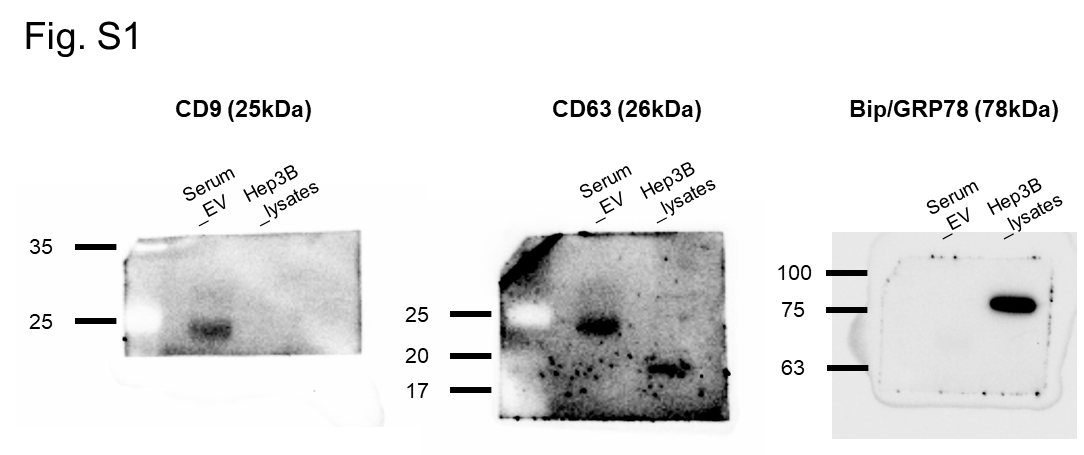


**Supplementary Figure S1. Full-length, uncropped western blot images corresponding to Fig. 1E.** Western blot analyses were performed using EVs isolated from serum and Hep3B cell lysates. The membranes show unedited bands, including molecular weight markers and all sample lanes.


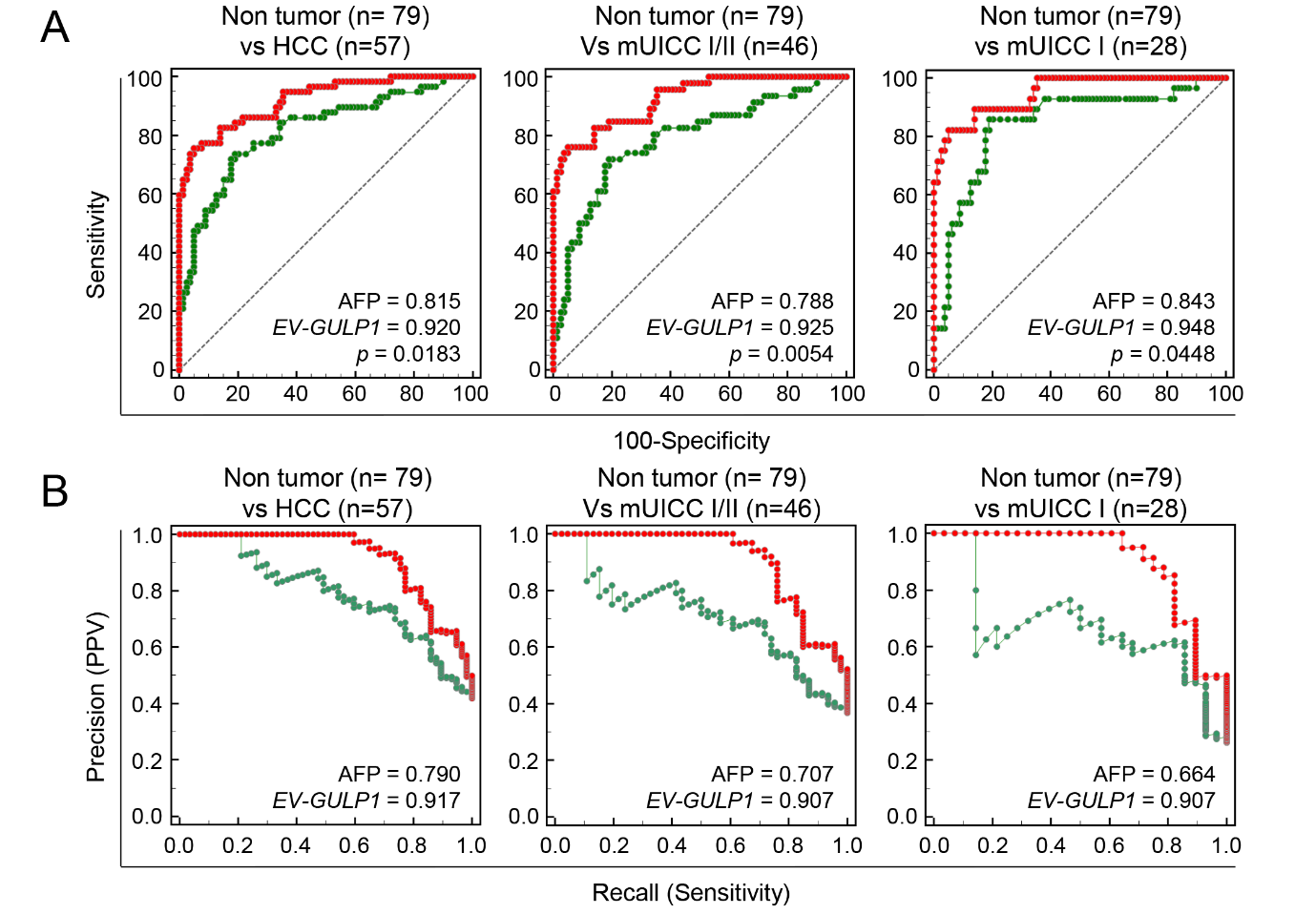


**Supplementary Figure S2. Diagnostic performance of small extracellular vesicle encapsulated *GULP1* (*EV-GULP1*) compared with alpha-fetoprotein (AFP).** (**A**) Receiver operating characteristic (ROC) curve analysis comparing the diagnostic accuracy of *EV-GULP1* and AFP in differentiating non-HCC (healthy controls [HC], chronic hepatitis [CH], and liver cirrhosis [LC]) from HCC. *EV-GULP1* consistently exhibited higher area under the curve (AUC) values than AFP, especially in early-stage HCC. Patients with missing AFP values were excluded from the analysis. (**B**) Precision-recall (PR) curve analysis demonstrating that *EV-GULP1* provides superior classification performance compared with AFP. AFP-missing cases were excluded from all relevant analyses.

**
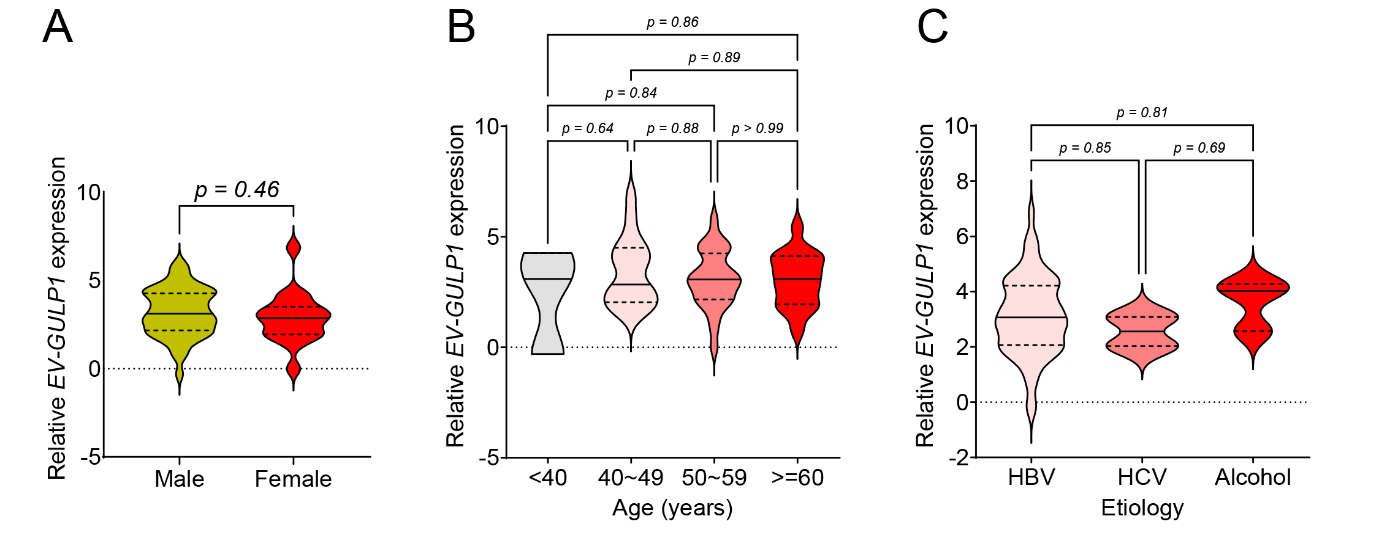
**

**Supplementary Figure S3. Subgroup analyses of serum-derived *EV-GULP1* expression by sex, age, and etiology.** (**A**) Violin plots showing relative *EV-GULP1* expression by sex; no significant difference was observed between male and female patients. (**B**) *EV-GULP1* expression across age strata (<40, 40–49, 50–59, ≥60 years) showing no significant between-group differences. (**C**) *EV-GULP1* expression by underlying disease etiology (HBV, HCV, alcohol-related), likewise demonstrating no significant differences. Comparisons were assessed using an unpaired Welch’s t-test (sex) and one-way ANOVA with Tukey’s post-hoc test (age and etiology). HBV, hepatitis B virus; HCV, hepatitis C virus.

**
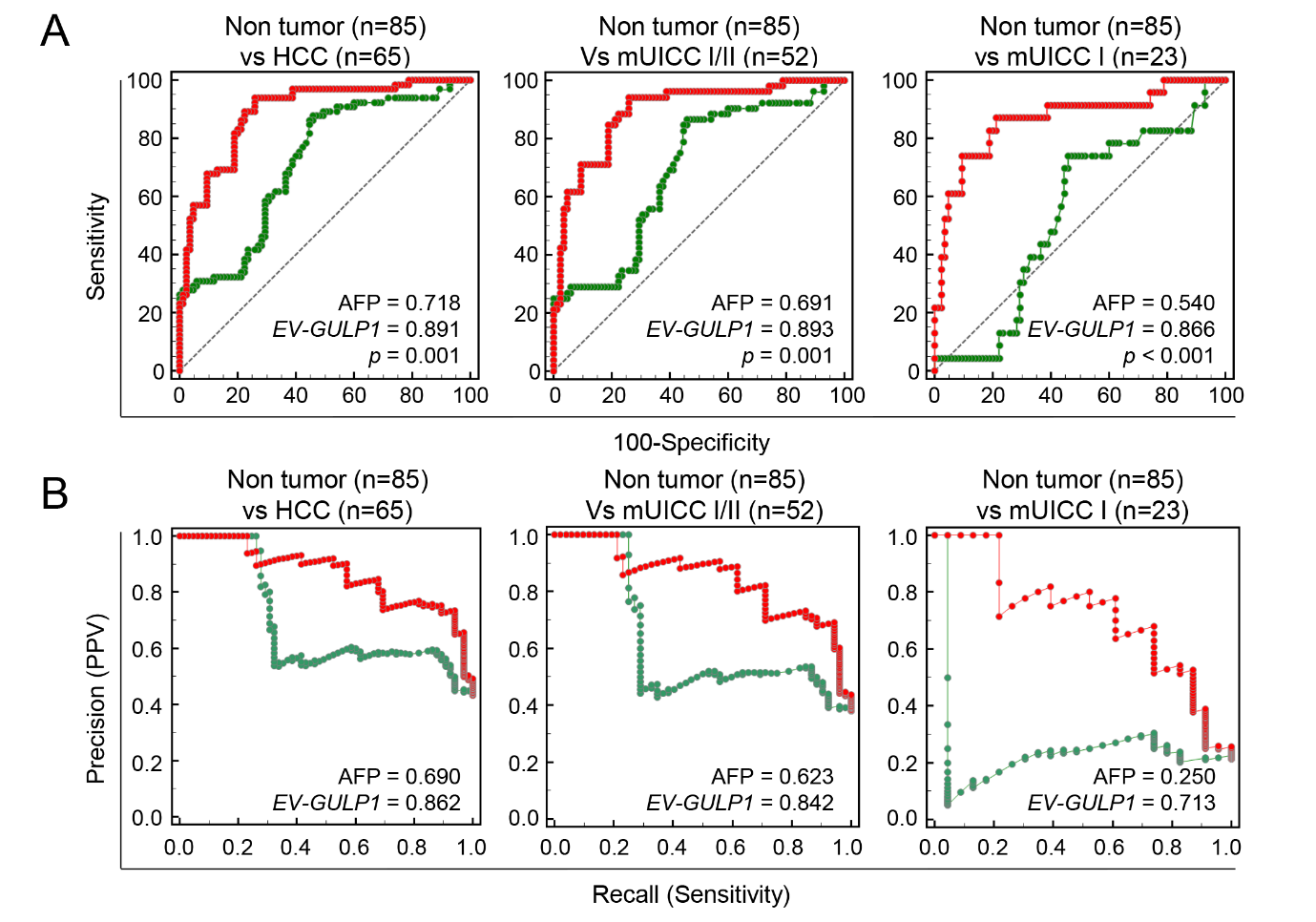
**

**Supplementary Figure. S4. External investigation of *EV-GULP1* diagnostic performance in a validation cohort.** (**A**) ROC curve analysis comparing the diagnostic accuracy of *EV-GULP1* and AFP in differentiating non-HCC (HC, CH, and LC) from HCC. *EV-GULP1* consistently exhibited higher area under the curve values than AFP, particularly in early-stage HCC. Those with missing AFP levels were not considered in the final analysis. (**B**) PR curve analysis showing *EV-GULP1*’s superior classification performance compared to AFP across all HCC stages. Patients with missing AFP values were excluded from the analysis.
